# Spatiotemporal dynamics of water and sediment quality under multi-source pollution: A case study in the Jinjing Watershed, China

**DOI:** 10.1371/journal.pone.0336027

**Published:** 2025-11-13

**Authors:** Lingling Tong, Feng Liu, Fatimah Md. Yusoff, Ahmad Zaharin Aris, Ahmad Fikri Abdullah, Yam Sim Khaw, Hui Teng Tan, Dejun Li, Murni Karim

**Affiliations:** 1 Department of Primary Education, Changsha Normal University, Changsha, Hunan, China; 2 International Institute of Aquaculture and Aquatic Science, Universiti Putra Malaysia, Port Dickson, Malaysia; 3 Institute of Subtropical Agriculture, Chinese Academic of Sciences, Changsha, Hunan, China; 4 Department of Aquaculture, Faculty of Agriculture, Universiti Putra Malaysia, Selangor, Malaysia; 5 Aquatic Animal Health and Therapeutics Laboratory, Institute of Bioscience, Universiti Putra Malaysia, Selangor, Malaysia; University of Mpumalanga, SOUTH AFRICA

## Abstract

The dynamics of physicochemical properties within rivers are essential for understanding the health and functioning of aquatic ecosystems. This study investigated the spatial and seasonal variability of water quality in both water and sediment phases across rivers with different pollution sources in the Jinjing Basin: Tuojia River (TR), Tuojia River substream (TRS) (farmland), Guojia River (GR), Guojia River substream (GRS) (woodlands) and Jinjing River (JR) (residential). Samples were collected during wet and dry seasons and analyzed using multivariate statistical approaches. Farmland-dominated rivers (TR and TRS) exhibited the highest nutrient concentrations in both water and sediment phases, with elevated nutrients, soil organic matter (SOM), and dissolved organic carbon (DOC), driven by fertilizer runoff and organic inputs. In contrast, woodland rivers (GR and GRS) displayed the lowest nutrient levels, benefiting from dense vegetation and natural nutrient retention processes. Seasonal variability revealed higher nutrient concentrations in the water phase and increased levels of ammonium nitrogen (NH_4_^+^-N) and SOM in the sediment phase during the wet season. In the dry season, reduced flow enhanced photosynthesis, resulting in higher pH and dissolved oxygen levels in the water phase and elevated pH and DOC in sediment. Principal component analysis further confirmed that nutrient pollution is predominantly influenced by agricultural runoff during the wet season, while reduced runoff in the dry season allowed natural processes to dominate. The findings underscore the importance of managing nutrient loads in both water and sediment, especially in farmland areas to ensure the sustainability of water resource management in the Jinjing Basin.

## Introduction

The health and functioning of aquatic ecosystems depend heavily on the dynamics of physicochemical properties within rivers. These properties such as nutrient concentrations, pH, dissolved oxygen (DO) and organic matter levels, can significantly influence the biological and chemical processes that sustain water quality. Understanding how these properties vary spatially and seasonally, especially across rivers with diverse pollution sources, is crucial for assessing the impact of human activities and natural processes on aquatic environments. In particular, river systems within agricultural, woodland and residential areas are subject to varying levels of nutrient pollution, which can alter water quality and ecosystem health [1]. Therefore, understanding these variations is essential for the effective management and conservation of water resources [2,3]. Moreover, the relationships among the physicochemical parameters are tightly interlinked through complex biological and chemical processes. These interactions differ across various land use types and seasons, highlighting the importance of examining not only the individual concentrations of pollutants, but also how their combined effects on river ecosystem health.

Numerous studies have underscored how land use and seasonal changes determine riverine water quality across different aquatic environments. For example, nutrient loads in agricultural catchments are often increased due to fertilizer runoff and livestock waste, triggering eutrophication and subsequent degradation of water quality [4]. Seasonality introduces another layer of complexity, especially in subtropical monsoon climates where the wet season promotes large quantities of nutrients via surface runoff, while the dry season typically facilitates biological activity and photosynthesis in aquatic systems [5]. Despite the growing body of research, many studies have examined either water or sediment alone, or have been limited to a single season or land-use type. Few have comprehensively addressed the spatiotemporal variability of multiple pollution sources across both water and sediment, particularly within the subtropical regions of China.

Jinjing Basin in China represents a diverse and dynamic watershed system influenced by varying land uses, including farmland, residential areas and woodlands. These land use patterns contribute to distinct pollution sources, such as agricultural runoff, domestic discharges and natural nutrient inputs, making the basin an ideal study site for understanding the interplay between human activities and river health [6]. Examining both water and sediment phases provides a comprehensive understanding of nutrient dynamics, as sediments serve as both sinks and sources of nutrients [7]. Seasonal variations further complicate these dynamics, with wet and dry periods altering nutrient transport and geochemical processes [8].

Multivariate approaches such as principal component analysis (PCA) and cluster analysis have been successfully applied in river systems to identify spatial and seasonal variations in pollution patterns. For instance, Khan et al. (2017) [9] utilized these tools to detect physicochemical and heavy metal pollution across land-use gradients in the Ramganga River. In addition, Ding et al. (2015) [6] demonstrated seasonal influence of small tributaries on the hydrochemistry of the main river channel within the basin. These studies support the validity of the methodological approach of the current study and illustrate the importance of examining the interactions between land use and seasonal dynamics in the Jinjing watershed.

Previous studies have demonstrated that various pollution sources and seasonal changes can significantly impact the water quality of water bodies. Agricultural activities, urban development and deforestation are key contributors to water pollution, often leading to a reduction in water quality [10,11]. Ding et al. (2015) [6] found that different land use patterns have varying consequences on the water supply environment in Dongjiang Basin based on a combination of field investigations and multivariate statistical analyses. Until now, only one study has reported on the land use and cover changes in the upper Jinjing River catchment from 1933 to 2005 [7]. However, there is limited research on the physicochemical properties of rivers within the Jinjing Basin, particularly regarding their spatial and seasonal variability and the influence of different pollution sources. While many studies have addressed spatial or temporal variability in river systems, relatively few have examined both water and sediment quality. In addition, there is limited research reporting how diverse land use types influence riverine biogeochemistry in subtropical watersheds. Thus, this leaves a gap in understanding how physicochemical properties co-vary in response to land use and seasonal hydrology within these complex ecosystems. To address this gap, this study aimed to (1) characterize the spatial and seasonal variability physicochemical parameters in both water and sediment across rivers subjected to various pollution sources within the Jinjing Basin; (2) examine the relationships among these parameters using multivariate statistical approach; and (3) identify pollution hotspots and provide appropriate environmental management recommendations. Understanding these variations will provide valuable insights for improving water quality management and ensuring the sustainability of aquatic ecosystems in the basin.

## Materials and methods

### Description of the study area

**Fig 1:**
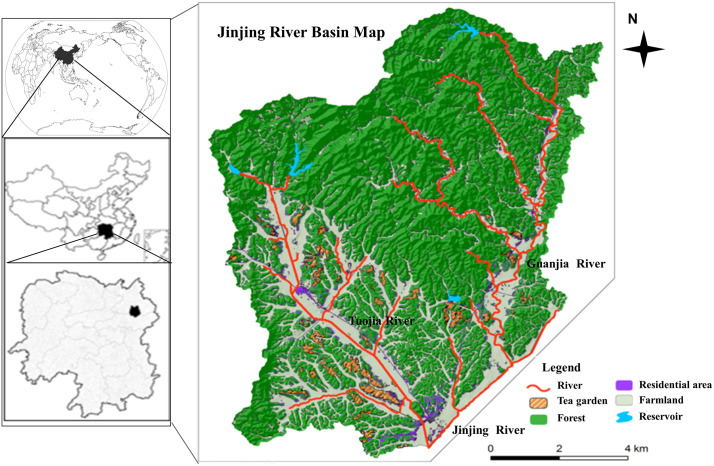
The map of the study area. The base map was created by the authors using public domain data from the USGS National Map (http://viewer.nationalmap.gov/viewer/).

This study focused on the Jinjing Basin (27°55’ ~ 28°40’N, 112°56’ ~ 113°30’), located in the upstream section of the Xiang Jiang River (Fig 1). The study area experiences a subtropical monsoon climate and is characteristic of the hilly land from typical of the southern Yangtze River region. The average annual temperature is 17.2 °C, with the average annual rainfall exceeding 1400 mm. Most of the rainfall occurs between April and June, accounting for 76% of the total annual rainfall. The average altitude in the basin is 98.3 m and the terrain is gently undulating with slopes ranging from 12.3% to 25.7%. The watershed is primarily composed of woodland, farmland and tea and vegetable fields. The farmland along the riverbanks is densely cultivated with frequent farming activities, while residential areas are also concentrated near the river. Improper fertilization practices and nitrogen pollution in the farmland are prominent environmental concerns in the area.

**Fig 2:**
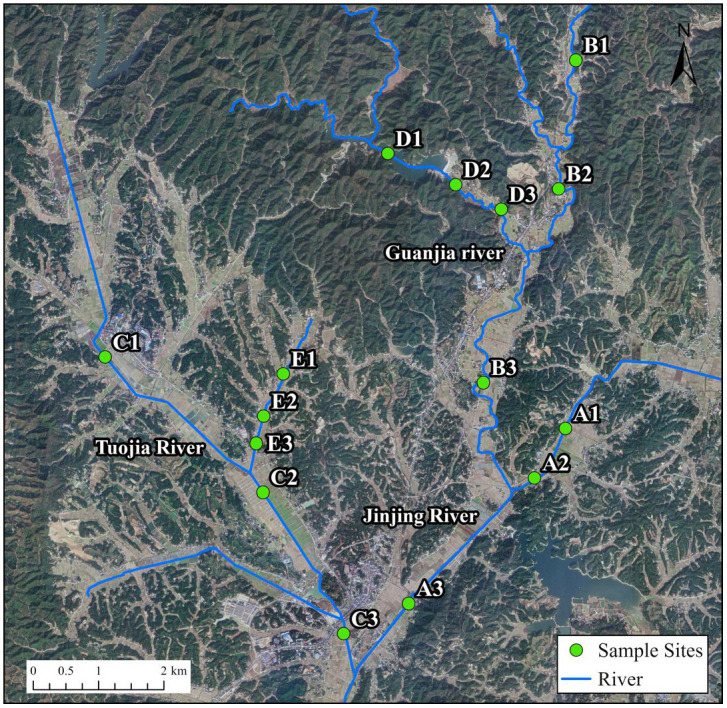
The map of the sampling points. The base map was created by the authors using public domain data from the USGS National Map (http://viewer.nationalmap.gov/viewer/).

Three tributaries with distinct pollution sources were selected for this study: (1) the Jinjing River (JR), influenced by residential areas (Sampling points: A1, A2 and A3); (2) the Guanjia River (GR) (Sampling points: B1, B2 and B3) and its substream (GRS) (Sampling points: D1, D2 and D3), representing woodland or mountain rivers; (3) the Tuojia River (TR) (Sampling points: C1, C2 and C3) and its substream (TRS) (Sampling points: E1, E2 and E3), predominantly surrounded by farmland. Sampling sites were established in the upper, middle and lower reaches of each river and their tributaries to capture spatial variability (Fig 2). Based on geographic information and field survey results, sampling points were strategically chosen to comprehensively cover areas affected by farmland runoff, urban drainage and other non-point sources. Additionally, the locations and environmental characteristics of the sampling sites were documented for both dry and wet seasons to ensure the representation of seasonal variations (Table 1). The research was conducted in a public river basin and therefore did not require a permit.

**Table 1:** Locations and environmental characteristics of sampling sites in two different seasons.

River	Area	Longitude	Latitude	Dry season			Wet season		
				Wide (m)	Flow rate (ms^-1^)	Temperature (°C)	Wide (m)	Flow rate (ms^-1^)	Temperature (°C)
Jinjing River	Residential area	113.39	28.54	14.33 ± 1.04	0.21 ± 0.04	11.47 ± 0.15	14.33 ± 1.04	0.27 ± 0.01	19.40 ± 0.26
Tuojia River	Farmland	113.37	28.56	4.56 ± 0.34	0.15 ± 0.03	11.33 ± 0.31	4.50 ± 0.22	0.19 ± 0.09	18.57 ± 0.42
Toujia River substream	Farmland	113.22	28.33	3.26 ± 0.25	0.35 ± 0.08	11.83 ± 0.45	3.40 ± 0.17	0.39 ± 0.11	18.27 ± 0.15
Guanjia River	Woodland	113.39	28.56	4.92 ± 0.77	0.24 ± 0.11	11.47 ± 0.29	4.94 ± 0.77	0.52 ± 0.24	17.53 ± 2.01
Guanjia River substream	Woodland	113.39	28.54	3.63 ± 0.76	0.36 ± 0.07	11.23 ± 0.06	3.67 ± 0.72	0.37 ± 0.06	18.40 ± 0.26

### Water and sediment sample collection

#### Water sample collection and field measurements.

Water samples were collected during December 2021 (dry season) and April 2022 (wet season) using 200 mL polyvinyl plastic bottles at the designated sampling sites (Fig 2). The samples were filtered by 0.45 µm filter membrane and stored at 4 °C within 2 days of collection to preserve their integrity for subsequent determination and analysis of relevant physical and chemical parameters.

At each sampling site, a portable water quality multi-parameter tester (SG68, Mettler-Toledo, Switzerland) was employed to measure river width, water temperature (T), pH and DO. Additionally, a current meter LS300-B, Wan-Xiang, China) was used to monitor the flow rate (V) in situ. The Oxidation-Reduction Potential (Eh) at each sampling site was measured using an ORP Meter (FJA-15, Teai-de, China) to provide a complete dataset for hydrological assessments.

#### Sediment collection and sample handling.

Sediment samples were collected concurrently with the water samples at each sampling site. A three-point sampling method was employed to collect the sediment (0–5 cm depth) from the riverbed using a grab sampler. The sediment was removed plant roots, fallen leaves and other residues, and passed through a 10 mesh sieve, then mixed and carefully placed into sterile zip-lock bags to prevent contamination. Each sediment was then divided into four portions for specific analyses: physicochemical properties, microbial activity, microbial gene abundance and sample retention for future use.

### Methods for determining physicochemical parameters in water and sediment

#### Water samples.

The parameters determined for the water samples included ammonium nitrogen (NH_4_^+^-N), nitrate nitrogen (NO_3_^-^-N), total nitrogen (TN) and total phosphorus (TP). The NH_4_^+^-N and NO_3_^-^-N were analyzed using a flow analyzer (AA3, German SEAL, China) and a total organic carbon analyzer (TOC-VWP, Shimadzu, Japan) after filtering the water sample through 0.45 µm membrane. The TN and TP were determined using a flow analyzer (AA3, German SEAL, China) following alkaline potassium persulfate and potassium persulfate digestions, respectively [12].

#### Sediment samples.

The sediment parameters analyzed included NH_4_^+^-N, NO_3_^-^-N, dissolved organic carbon (DOC), pH, TN and soil organic matter (SOM). The NH_4_^+^-N and NO_3_^-^-N were extracted from sediment using potassium sulfate, filtered and directly measured with a flow analyzer (AA3, German SEAL, China). The DOC was extracted with potassium sulfate, filtered and mixed in a 1:1 ratio with a neutral sodium hexametaphosphate solution. After shaking, DOC was measured using an organic carbon analyzer (TOC-VWP, Shimadzu, Japan). Sediment pH was determined using a Mettler-Toledo 320 pH meter, with a soil to water ratio of 2.5:1. Sediment TN was quantified using the semi-micro Kjeldahl method and analyzed with a flow analyzer (AA3, German SEAL, China) after digestion. The SOM was determined by titration with ferrous sulfate after treatment with concentrated sulfuric acid and potassium dichromate [13].

### Statistical analysis

The assumptions of homoscedasticity and normality were initially evaluated using Levene’s test for variance homogeneity and the Shapiro-Wilk test, respectively. The spatial and temporal distributions of physicochemical parameters in the water and sediment phase of the rivers within the Jinjing Basin were analyzed separately using two-way analysis of variance (ANOVA), followed by Tukey’s post hoc test for multiple comparisons. Additionally, a pair t-test was performed to evaluate the seasonal variation in the physicochemical properties of individual rivers across the dry and wet seasons. Moreover, a PCA was conducted separately on the spatial and temporal distributions of physicochemical parameters in both the water and sediment phase of the rivers within the Jinjing Basin to identify correlations among the variables and reveal possible trends in the data. Furthermore, inverse distance weighted (IDW) interpolation was performed using ArcGIS Pro (version 3.5, Esri Inc.) to visualize the spatial and temporal distributions of physicochemical parameters in both the water and sediment phase of the rivers within the Jinjing Basin. Data were presented as means ± standard deviation. All statistical analyses were performed using Minitab 17.1 (Minitab Inc., State College, Pennsylvania, USA) with differences considered significant at *p* < 0.05.

## Results

### The physicochemical parameters of water from Jinjing Basin

From the spatial perspective, the concentration of NH_4_^+^-N in TRS was the highest (0.76 ± 0.53 mg L^-1^) among the other rivers (Table 2). These concentrations were significantly higher (*p* < 0.05) than GR (0.15 ± 0.21 mg L^-1^) and GRS (0.16 ± 0.15 mg L^-1^). Among the studied rivers, the TRS exhibited the greatest concentration of NO_3_^-^-N, with values of 1.30 ± 0.65 mg L^-1^. This concentration was significantly higher (*p* < 0.05) than that observed in the GRS (0.44 ± 0.25 mg L^-1^). In comparison to other rivers, the TRS also demonstrated the highest concentration of TN (3.33 ± 1.46 mg L^-1^). This concentration was significantly higher (*p* < 0.05) than JR (1.57 ± 0.21 mg L^-1^), GR (1.19 ± 0.38 mg L^-1^) and GRS (0.70 ± 0.15 mg L^-1^). For TP, GRS showed the lowest concentration (0.05 ± 0.01 mg L^-1^) among the other rivers. This concentration was significantly lower (*p* < 0.05) compared to TR (0.15 ± 0.04 mg L^-1^) and TRS (0.18 ± 0.10 mg L^-1^). Meanwhile, GR illustrated the greatest pH value (7.60 ± 0.23) in comparison to the other rivers. This value was significantly higher (*p* < 0.05) than TRS (7.05 ± 0.80). Guanjia River showed the lowest Eh value (−32.74 ± 14.19 mV) among the rivers in this study, while the greatest Eh was found in TRS (−15.05 ± 3.54 mV). The Eh values of both GR and GRS (−32.59 ± 2.43 mV) were significantly lower (*p* < 0.05) than the other rivers in the present study. The highest DO concentrations were observed in the GRS (8.13 ± 1.46 mg L^-1^), followed by the TRS (7.64 ± 1.49 mg L^-1^), JR (7.62 ± 1.20 mg L^-1^), GR (7.55 ± 1.33 mg L^-1^) and TR (7.23 ± 1.12 mg L^-1^) with no significant differences (*p* > 0.05) between them.

**Table 2:** Spatial and temporal variability of physicochemical parameters in water samples from different rivers in the Jinjing Basin.

Main effect		Physicochemical water parameters						
River	Season	NH4^ + ^-N (mg L^-1^)	NO3^—^N (mg L^-1^)	TN (mg L^-1^)	TP (mg L^-1^)	pH	DO (mg L^-1^)	Eh (mV)
JR	Wet	0.17	1.10	1.57	0.14	7.25^a^	6.67	−16.63^ab^
	Dry	0.29	0.89	1.56	0.10	7.69^a^	8.57	−25.47^abc^
GR	Wet	0.03	0.84	1.16	0.07	7.43^a^	6.43	−21.77^ab^
	Dry	0.27	0.53	1.21	0.08	7.76^a^	8.67	−43.70^c^
GRS	Wet	0.12	0.30	0.72	0.04	7.14^a^	6.83	−31.97^bc^
	Dry	0.20	0.57	0.68	0.05	7.70^a^	9.43	−33.20^bc^
TR	Wet	0.44	1.17	2.47	0.15	7.08^ab^	6.23	−17.93^ab^
	Dry	0.30	0.92	1.66	0.14	7.31^a^	8.23	−18.93^ab^
TRS	Wet	0.69	1.50	3.45	0.18	6.37^b^	6.37	−18.27^ab^
	Dry	0.82	1.10	3.20	0.18	7.73^a^	8.90	−11.83^a^
Pool SEM		0.18	0.23	0.46	0.03	0.15	0.30	3.85
**Mean river**								
JR		0.23^ab^	1.00^ab^	1.57^b^	0.12^abc^	7.47^ab^	7.62	−21.05^a^
GR		0.15^b^	0.69^ab^	1.19^b^	0.08^bc^	7.60^a^	7.55	−32.74^b^
GRS		0.16^b^	0.44^b^	0.70^b^	0.05^c^	7.42^ab^	8.13	−32.59^b^
TR		0.37^ab^	1.05^ab^	2.07^ab^	0.15^ab^	7.20^ab^	7.23	−18.43^a^
TRS		0.76^a^	1.30^a^	3.33^a^	0.18^a^	7.05^b^	7.64	−15.05^a^
**Mean season**								
Wet		0.29	0.98	1.87	0.12	7.05^b^	6.51^b^	−21.31^a^
Dry		0.38	0.80	1.66	0.11	7.64^a^	8.76^a^	−26.63^b^
**Probability**								
River		0.020	0.014	0.000	0.003	0.014	0.091	0.041
Season		0.473	0.249	0.475	0.773	0.000	0.000	0.000
River x season		0.888	0.657	0.882	0.927	0.010	0.706	0.017

JR, Jinjing River; GR, Guanjia River; GRS, Guanjia River substream; TR, Tuojia River; TRS, Tuojia River substream; NH_4_^+^-N, ammonium nitrogen; NO_3_^-^-N, nitrate nitrogen; TN, total nitrogen; TP, total phosphorus; DO, dissolved oxygen; Eh, redox potential. Different letter within the same column and section indicate significant difference (*p* < 0.05).

With respect to time, the DO and pH values during the dry season (8.76 ± 0.61 mg L^-1^; 7.64 ± 0.30) were significantly higher (*p* < 0.05) than those observed during wet season (6.51 ± 0.48 mg L^-1^; 7.05 ± 0.42) across all rivers. In contrast, the Eh value during the wet season (−21.31 ± 7.79 mV) was significantly higher (*p* < 0.05) than that of dry season (−26.63 ± 12.96 mV) in all studied rivers. For the remaining physicochemical water parameters such as NO_3_ ⁻ -N, TN and TP, the values were greater during the wet season compared to the dry season, with the exception of NH_4_^+^-N. However, none of these differences between seasons were statistically significant (*p* > 0.05).

The interaction between river and season revealed that the chemical parameters including NH_4_^+^-N (ranged from 0.30 to 0.82 mg L^-1^), NO_3_^-^-N (ranged from 0.92 to 1.17 mg L^-1^), TN (ranged from 1.66 to 3.45 mg L^-1^) and TP (ranged from 0.14 to 0.18 mg L^-1^) were consistently higher in TR and TRS during both seasons compared to the other rivers. Conversely, GR and GRS exhibited the lowest concentrations of NH_4_^+^-N (ranged from 0.03 to 0.27 mg L^-1^), NO_3_^-^-N (ranged from 0.30 to 0.84 mg L^-1^), TN (ranged from 0.68 to 1.21 mg L^-1^) and TP (ranged from 0.04 to 0.08 mg L^-1^) in the respective season among the investigated rivers. No apparent trends were observed for pH, DO and Eh in the interaction between river and season.

### The physicochemical parameters of sediment from Jinjing Basin

Geographically, the sediment from TR and TRS exhibited the highest concentrations of TN (1.84 ± 0.19 g L^-1^; 2.52 ± 0.54 mg L^-1^), SOM (35.89 ± 4.33 g kg^-1^; 44.43 ± 13.16 g kg^-1^) and DOC (86.92 ± 20.66 mg kg^-1^; 86.54 ± 70.32 mg kg^-1^) among the studied rivers (Table 3). These values were significantly higher (*p* < 0.05) than those observed in GR (TN: 0.99 ± 0.44 g L^-1^; SOM: 21.46 ± 4.75 g kg^-1^; DOC: 42.67 ± 30.89 mg kg^-1^). In contrast, the sediment pH in TR (6.24 ± 0.26) and TRS (6.17 ± 0.24) was the lowest compared to the other rivers. The sediment pH in TRS was also found to be significantly lower (*p* < 0.05) than in the JR (6.94 ± 0.49) and GR (6.92 ± 0.48). On the other hand, sediment from TR and TRS showed the highest concentrations of NH_4_^+^-N (62.68 ± 15.45 mg L^-1^; 58.60 ± 14.11 mg L^-1^) with no significant differences (*p* > 0.05) in comparison to the investigated rivers. Meanwhile, the highest concentration of NO_3_ ⁻ -N was identified in the sediment from JR (0.39 ± 0.24 mg L^-1^), though this was not significantly different (*p* > 0.05) from the concentrations observed in the other rivers.

**Table 3:** Spatial and temporal variability of physicochemical parameters in sediment samples from different rivers in the Jinjing Basin.

Main effect		Physicochemical water parameters					
River	Season	NH^4 + ^-N (mg kg^-1^)	NO_3_-N (mg kg^-1^)	TN (g kg^-1^)	SOM (g kg^-1^)	DOC (mg kg^-1^)	pH
JR	Wet	48.16	0.38	1.35	22.92	23.36^bc^	7.03
	Dry	32.32	0.40	1.81	27.91	77.22^b^	6.85
GR	Wet	54.09	0.35	1.12	25.42	18.31^bc^	6.79
	Dry	24.11	0.11	0.87	17.50	67.02^bc^	7.04
GRS	Wet	49.87	0.22	1.49	25.58	12.80^c^	6.29
	Dry	29.29	0.05	1.52	21.19	49.74^bc^	6.49
TR	Wet	60.30	0.16	1.78	38.01	20.06^bc^	6.15
	Dry	56.91	0.53	1.89	33.76	153.78^a^	6.33
TRS	Wet	69.53	0.10	2.70	48.98	26.05^bc^	6.05
	Dry	55.83	0.32	2.34	39.87	147.02^a^	6.29
Pool SEM		9.14	0.19	0.25	4.59	12.26	0.26
**Mean river**							
JR		40.24	0.39	1.58^bc^	25.41^bc^	50.29^ab^	6.94^a^
GR		39.10	0.23	0.99^c^	21.46^c^	42.67^b^	6.92^a^
GRS		39.58	0.13	1.50^bc^	23.38^bc^	31.27^b^	6.39^ab^
TR		58.60	0.35	1.84^ab^	35.89^ab^	86.92^a^	6.24^ab^
TRS		62.68	0.21	2.52^a^	44.43^a^	86.54^a^	6.17^b^
**Mean season**							
Wet		56.39^a^	0.24	1.69	32.18	20.12^b^	6.46
Dry		39.69^b^	0.28	1.69	28.04	98.96^a^	6.60
**Probability**							
River		0.058	0.644	0.000	0.000	0.000	0.006
Season		0.009	0.725	0.993	0.169	0.000	0.408
River x season		0.690	0.450	0.541	0.583	0.001	0.910

JR, Jinjing River; GR, Guanjia River; GRS, Guanjia River substream; TR, Tuojia River; TRS, Tuojia River substream; NH_4_^+^-N, ammonium nitrogen; NO_3_^-^-N, nitrate nitrogen; TN, total nitrogen; SOM, soil organic matter; DOC, dissolved organic carbon. Different letter within the same column and section indicate significant difference (*p* < 0.05).

Across seasons, sediment in the wet season (56.39 ± 13.80 mg L^-1^) displayed significantly higher (*p* < 0.05) concentrations of NH_4_^+^-N than in the dry season (39.69 ± 20.95 mg L^-1^). In contrast, a significantly higher (*p* < 0.05) DOC concentration was identified in the sediment during the dry season (98.96 ± 50.78 mg kg^-1^) compared to the wet season (20.12 ± 8.09 mg kg^-1^). For the rest of physicochemical water parameters such as NO_3_ ⁻ -N and pH, the values were higher in the sediment during the dry season (0.28 ± 0.41 mg L^-1^; 6.60 ± 0.52) than in the wet season (0.24 ± 0.17 mg L^-1^; 6.46 ± 0.52), though the differences were not significant (*p* > 0.05). Remarkably, the same value of TN (1.69 mg L^-1^) was acquired for the sediment for both the wet and dry season.

The interaction between river and season indicated DOC concentrations were significantly higher (*p* < 0.05) in the sediment from TR (153.78 ± 27.01 mg kg^-1^) and its substream (147.02 ± 57.60 mg kg^-1^) during the dry season compared to the other rivers. Additionally, the sediment from TR and its tributary displayed higher concentrations of NH_4_^+^-N (ranged from 55.83 to 69.53 mg L^-1^), TN (ranged from 1.78 to 2.70 g L^-1^) and SOM (ranged from 38.01 to 48.98 g kg^-1^) during both seasons than the studied rivers, although the differences were not significant (*p* > 0.05). Conversely, lower pH values (ranged from 6.05 to 6.33) were found in the sediment from TR and TRS during both seasons compared to other rivers with no significant differences (*p* > 0.05). For NO_3_^-^-N, no clear pattern was observed for the interaction between river sediment and season.

### Physicochemical indexes of water phase across different rivers during dry and wet seasons

To evaluate the seasonal impact on individual rivers within the Jinjing Basin, a paired t-test was conducted. The results revealed that both the TR and TRS exhibited higher pH and DO levels during the dry season (pH: 7.31 ± 0.22 to 7.73 ± 0.45; DO: 8.23 ± 0.38 to 8.90 ± 0.56 mg L^-1^) compared to the wet season (pH: 6.37 ± 0.12 to 7.08 ± 0.35; DO: 6.23 ± 0.06; 6.37 ± 0.67 mg L^-1^) (Table 4). A significant difference (*p* < 0.05) was observed in DO levels between the seasons for these rivers, while only TRS showed significantly higher (*p* < 0.05) pH level during the dry season compared to the wet season. Conversely, the concentrations of NO_3_ ⁻ -N and TN were higher during the wet season (NO_3_ ⁻ -N: 1.17 ± 0.07 to 1.50 ± 0.86 mg L^-1^; TN: 2.47 ± 0.68 to 3.45 ± 2.20 mg L^-1^) compared to the dry season (NO_3_ ⁻ -N: 0.92 ± 0.30 to 1.10 ± 0.43 mg L^-1^; TN: 1.66 ± 0.46 to 3.20 ± 0.66 mg L^-1^) for these rivers. No clear seasonal patterns were identified for NH_4_^+^-N, TP or Eh values in these rivers.

**Table 4:** Physicochemical parameters of water phase in individual river of Jinjing basin during wet and dry seasons.

River	NH^4 + ^-N (mg L^-1^)		NO_3_-N (mg L^-1^)		TN (mg L^-1^)		TP (mg L^-1^)		pH		DO (mg L^-1^)		Eh (mV)	
	Wet	Dry	Wet	Dry	Wet	Dry	Wet	Dry	Wet	Dry	Wet	Dry	Wet	Dry
Jinjing River	0.17 ± 0.14^a^	0.29 ± 0.17^a^	1.10 ± 0.06^a^	0.89 ± 0.03^a^	1.57 ± 0.24^a^	1.56 ± 0.24^a^	0.14 ± 0.02^a^	0.10 ± 0.03^a^	7.25 ± 0.31^a^	7.69 ± 0.40^a^	6.67 ± 0.38^b^	8.57 ± 0.85^a^	−16.63 ± 10.64^a^	−25.47 ± 5.29^a^
Guanjia River	0.03 ± 0.01^b^	0.27 ± 0.26^a^	0.84 ± 0.59^a^	0.53 ± 0.42^b^	1.16 ± 0.46^a^	1.21 ± 0.39^a^	0.07 ± 0.05^a^	0.08 ± 0.01^a^	7.43 ± 0.15^a^	7.76 ± 0.16^b^	6.43 ± 0.74^b^	8.67 ± 0.35^a^	−21.77 ± 8.17^a^	−43.70 ± 8.70^b^
Guanjia River substream	0.12 ± 0.16^a^	0.20 ± 0.17^a^	0.30 ± 0.16^a^	0.57 ± 0.27^a^	0.72 ± 0.20^a^	0.68 ± 0.11^a^	0.04 ± 0.00^a^	0.05 ± 0.00^a^	7.14 ± 0.07^a^	7.70 ± 0.09^b^	6.83 ± 0.35^b^	9.43 ± 0.35^a^	−31.97 ± 2.86^a^	−33.20 ± 2.33^a^
Tuojia River	0.44 ± 0.39^a^	0.30 ± 0.13^a^	1.17 ± 0.07^a^	0.92 ± 0.30^a^	2.47 ± 0.68^a^	1.66 ± 0.46^b^	0.15 ± 0.02^a^	0.14 ± 0.06^a^	7.08 ± 0.35^a^	7.31 ± 0.22^a^	6.23 ± 0.06^b^	8.23 ± 0.38^a^	−17.93 ± 1.40^a^	−18.93 ± 12.02^a^
Tuojia River substream	0.69 ± 0.60^a^	0.82 ± 0.57^a^	1.50 ± 0.86^a^	1.10 ± 0.43^a^	3.45 ± 2.20^a^	3.20 ± 0.66^a^	0.18 ± 0.15^a^	0.18 ± 0.05^a^	6.37 ± 0.12^b^	7.73 ± 0.45^a^	6.37 ± 0.67^b^	8.90 ± 0.56^a^	−18.27 ± 0.15^b^	−11.83 ± 0.45^a^

NH_4_^+^-N, ammonium nitrogen; NO_3_^-^-N, nitrate nitrogen; TN, total nitrogen; TP, total phosphorus; DO, dissolved oxygen; Eh, redox potential. Different letter within the same row (wet and dry) and section indicate significant difference (*p* < 0.05).

For JR, the levels of NO_3_^-^-N, TN, TP and Eh were higher during the wet season (NO_3_ ⁻ -N: 1.10 ± 0.06 mg L^-1^; TN: 1.57 ± 0.24 mg L^-1^; TP: 0.14 ± 0.02 mg L^-1^; Eh: −16.63 ± 10.64 mV) compared to the dry season (NO_3_^-^-N: 0.89 ± 0.03 mg L^-1^; TN: 1.56 ± 0.24 mg L^-1^; TP: 0.10 ± 0.03 mg L^-1^; Eh: −25.47 ± 5.29 mV). In contrast, JR displayed greater values of NH_4_^+^-N and pH during the dry season (NH_4_^+^-N: 0.29 ± 0.17 mg L^-1^; pH: 7.69 ± 0.40) compared to wet season (NH_4_^+^-N: 0.17 ± 0.14 mg L^-1^; pH: 7.25 ± 0.31). Furthermore, only DO level of this river was significantly higher (*p* < 0.05) during the dry season than during wet season.

Guanjia River and GRS demonstrated greater levels of NH_4_^+^-N, TP, pH and DO during the dry season (NH_4_^+^-N: 0.20 ± 0.17 to 0.27 ± 0.26 mg L^-1^; TP: 0.05 ± 0.00 to 0.08 ± 0.01 mg L^-1^; pH: 7.70 ± 0.09 to 7.76 ± 0.16; DO: 8.67 ± 0.35 to 9.43 ± 0.35 mg L^-1^) compared to the wet season (NH_4_^+^-N: 0.03 ± 0.01 to 0.12 ± 0.16 mg L^-1^; TP: 0.04 ± 0.00 to 0.07 ± 0.05 mg L^-1^; pH: 7.14 ± 0.07 to 7.43 ± 0.15; DO: 6.43 ± 0.74 to 6.83 ± 0.35 mg L^-1^). Additionally, the pH and DO levels of these rivers were significantly higher (*p* < 0.05) during the dry season than during the wet season. Moreover, only GR showed significantly higher (*p* < 0.05) NH_4_^+^-N concentration during the dry season compared to the wet season. Meanwhile, significantly higher (*p* < 0.05) values of NO_3_^-^-N and Eh were found exclusively in GR during wet season (NO_3_ ⁻ -N: 0.84 ± 0.59 mg L^-1^; Eh: −21.77 ± 8.17 mV) than during the dry season (NO_3_ ⁻ -N: 0.53 ± 0.59 mg L^-1^; Eh: −43.70 ± 8.70 mV). No apparent difference in TP concentrations were identified between seasons for these two rivers.

Considering all physicochemical parameters, a PCA was performed, consolidating the parameters from each of the two seasons into four distinct components (Table 5). Among these new variables, the first and second components (PC1 and PC2) with eigenvalues greater than 1, collectively accounted for at least 89% of the total variance for both wet and dry seasons. In the wet season, PC1 explained 77.11% of the total variance, showing the highest positive correlations with nutrient-related variables such as NH_4_^+^-N, NO_3_ ⁻ -N, TN and TP (Table 5). In contrast, PC2 that responsible for 16.07% of the total variance, exhibited stronger positive correlations with pH and Eh. Akin to wet season, PC1 in dry season remained highly associated with nutrient-related variables but accounted for a smaller proportion of the total variance at 65.44% (Table 5). On the other hand, PC2 in dry season explained a higher proportion of total variance, at 24.56%, demonstrating a higher positive correlation with pH and DO.

**Table 5:** Weight and eigenanalysis of physicochemical parameters in the water phase across different rivers during (A) wet and (B) dry season in the principal component analysis.

A.				
Variable	PC1	PC2	PC3	PC4
NH_4_^ + ^-N	0.391	−0.374	−0.125	0.467
NO_3_^-^-N	0.415	0.191	0.196	−0.509
TN	0.424	−0.153	−0.089	−0.051
TP	0.415	0.084	0.329	0.560
pH	−0.324	0.613	−0.063	0.446
DO	−0.326	−0.341	0.835	0.033
Eh	0.335	0.550	0.360	−0.082
Eigenvalue	5.398	1.125	0.425	0.052
Proportion	77.114	16.068	6.069	0.748
Cumulative	77.114	93.183	99.252	100.000
B.				
Variable	PC1	PC2	PC3	PC4
NH_4_^ + ^-N	0.398	0.376	−0.117	−0.394
NO_3_^-^-N	0.451	−0.037	0.155	0.574
TN	0.441	0.218	−0.215	−0.118
TP	0.458	−0.045	−0.165	−0.349
pH	−0.124	0.680	−0.422	0.486
DO	−0.185	0.586	0.672	−0.241
Eh	0.429	−0.035	0.509	0.293
Eigenvalue	4.581	1.719	0.537	0.163
Proportion	65.443	24.556	7.668	2.334
Cumulative	65.443	89.998	97.666	100.000

NH_4_^+^-N, ammonium nitrogen; NO_3_^-^-N, nitrate nitrogen; TN, total nitrogen; TP, total phosphorus; DO, dissolved oxygen; Eh, redox potential.

**Fig 3:**
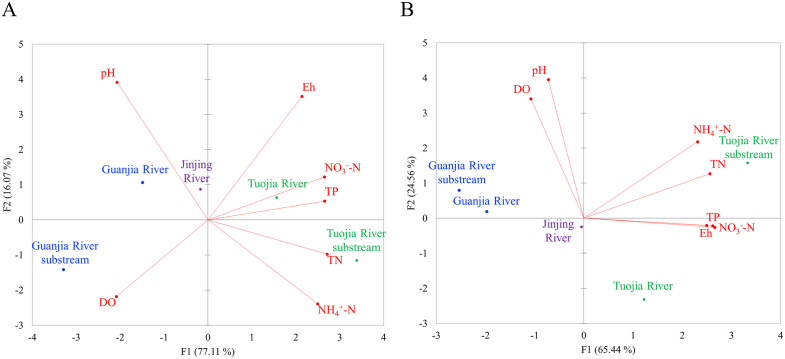
Principal Components Analysis (PCA) of physicochemical parameters in the water phase of rivers with Jinjing Basin during (A) the wet season and (B) the dry seasons. TP, total phosphorus, TN, total nitrogen, Eh, redox potential, DO, dissolved oxygen, NO_3_^-^-N, nitrate nitrogen and NH_4_^+^-N, ammonium nitrogen. Green colour indicates farmland, purple colour indicates residential area and blue colour indicates woodland. The base map was created by the authors using public domain data from the USGS National Map (http://viewer.nationalmap.gov/viewer/).

**Fig 4:**
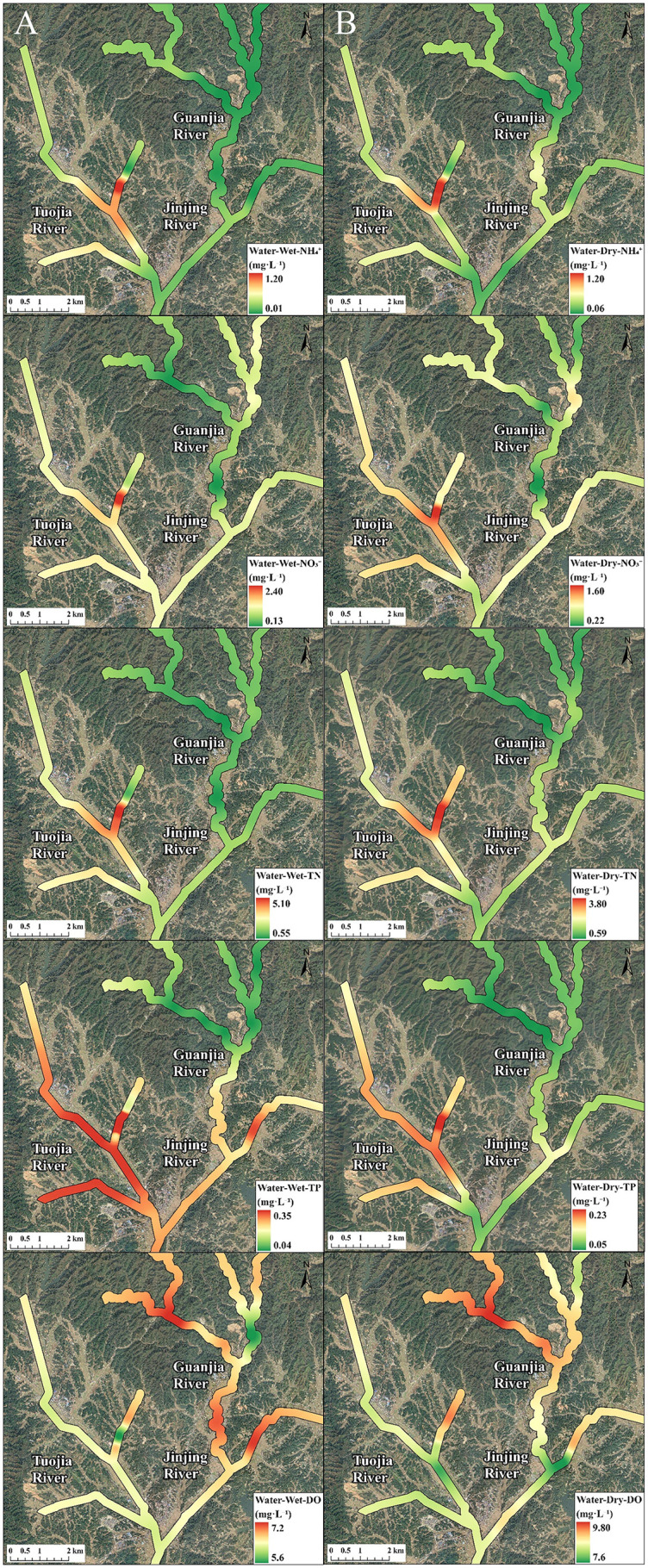
Inverse Distance Weighted (IDW) interpolation of physicochemical parameters in the water phase of rivers with Jinjing Basin during (A) the wet season and (B) the dry seasons. NH_4_^+^-N, ammonium nitrogen, NO_3_⁻-N, nitrate nitrogen, TN, total nitrogen, TP, total phosphorus and DO, dissolved oxygen. Red colour indicates the highest value of the parameters, while dark green indicates the lowest value of the parameters.The base map was created by the authors using public domain data from the USGS National Map (http://viewer.nationalmap.gov/viewer/).

The PCA biplot revealed that the water phases of five rivers within the Jinjing Basin were distinctly separated by the first two components, irrespective of the season (Fig 3). These rivers were further divided into two clusters based on PC1. The first cluster consisted of TR and TRS, showed elevated nutrient content and Eh values. On the other hand, the second cluster comprising GR, GRS and JR was characterized by higher pH and DO levels. No apparent differences in the PCA biplot based on seasonal differences except for the variation in pH and DO levels. These two parameters were closely associated in the dry season than the wet season. For instance, the water phases of the rivers in Jinjing Basin displayed higher pH and DO levels during the dry season than in the wet season. The spatiotemporal distribution patterns of physicochemical parameters in the water phase, as revealed by IDW interpolation, correspond well with the findings from PCA (Fig 4).

### Physicochemical indexes of sediment phase across different rivers during dry and wet seasons

A paired t-test was performed to assess how seasonal changes affect the sediment phase of individual rivers within the Jinjing Basin. The sediment characteristics varied across rivers and between seasons. Tuojia River and TRS revealed higher NH_4_^+^-N concentrations in the wet season (60.30 ± 15.87 to 69.53 ± 14.00 mg kg⁻¹) compared to the dry season (55.83 ± 10.93 to 56.91 ± 17.25 mg kg ⁻ ¹) with no significant different (*p* > 0.05) (Table 6). The value of SOM was slightly higher during the wet season (38.01 ± 18.08 to 48.98 ± 9.09 g kg ⁻ ¹) compared to the dry season (33.76 ± 11.15 to 39.87 ± 1.74 g kg ⁻ ¹). Conversely, NO_3_ ⁻ -N levels were generally higher in the dry season (0.32 ± 0.23 to 0.53 ± 0.90 mg kg ⁻ ¹) compared to the wet season (0.10 ± 0.04 to 0.16 ± 0.05 mg kg ⁻ ¹) with no significant differences (*p* > 0.05). Additionally, DOC concentrations were significantly higher (*p* < 0.05) in the dry season (147.02 ± 57.60 to 153.78 ± 27.01 mg kg ⁻ ¹) than in the wet season (20.06 ± 9.02 to 26.05 ± 3.92 mg kg ⁻ ¹), while pH values remained stable across seasons. The TN values did not exhibit specific trend between seasons.

**Table 6:** Physicochemical parameters of sediment phase in individual river of Jinjing basin during wet and dry seasons.

River	NH^4 + ^-N (mg kg^-1^)		NO_3_-N (mg kg^-1^)		TN (g kg^-1^)		SOM (g kg^-1^)		DOC (mg kg^-1^)		pH	
	Wet	Dry	Wet	Dry	Wet	Dry	Wet	Dry	Wet	Dry	Wet	Dry
Jinjing River	48.16 ± 13.64^a^	32.32 ± 32.06^a^	0.38 ± 0.27^a^	0.40 ± 0.26^a^	1.35 ± 0.12^a^	1.81 ± 0.32^a^	22.92 ± 2.87^a^	27.91 ± 6.54^a^	23.36 ± 9.70^b^	77.22 ± 5.22^a^	7.03 ± 0.19^a^	6.85 ± 0.74^a^
Guanjia River	54.09 ± 10.45^a^	24.11 ± 11.75^b^	0.35 ± 0.03^a^	0.11 ± 0.09^b^	1.12 ± 0.38^a^	0.87 ± 0.10^a^	25.42 ± 2.79^a^	17.50 ± 1.84^b^	18.31 ± 9.45^b^	67.02 ± 10.42^a^	6.79 ± 0.65^a^	7.04 ± 0.67^a^
Guanjia River substream	49.87 ± 11.60^a^	29.29 ± 6.78^a^	0.22 ± 0.15^a^	0.05 ± 0.03^a^	1.49 ± 0.22^a^	1.52 ± 0.21^a^	25.58 ± 2.78^a^	21.19 ± 4.98^a^	12.80 ± 4.90^b^	49.74 ± 4.38^a^	6.29 ± 0.23^a^	6.49 ± 0.30^a^
Tuojia River	60.30 ± 15.87^a^	56.91 ± 17.25^a^	0.16 ± 0.05^a^	0.53 ± 0.90^a^	1.78 ± 0.78^a^	1.89 ± 0.47^b^	38.01 ± 18.08^a^	33.76 ± 11.15^a^	20.06 ± 9.02^b^	153.78 ± 27.01^a^	6.15 ± 0.40^a^	6.33 ± 0.32^a^
Tuojia River substream	69.53 ± 14.00^a^	55.83 ± 10.93^a^	0.10 ± 0.04^a^	0.32 ± 0.23^a^	2.70 ± 0.78^a^	2.34 ± 0.40^a^	48.98 ± 9.09^a^	39.87 ± 1.74^a^	26.05 ± 3.92^b^	147.02 ± 57.60^a^	6.05 ± 0.34^a^	6.29 ± 0.21^a^

NH_4_^+^-N, ammonium nitrogen; NO_3_^-^-N, nitrate nitrogen; TN, total nitrogen; SOM, soil organic matter; DOC, dissolved organic carbon. Different letter within the same row (wet and dry) and section indicate significant difference (*p* < 0.05).

In JR, NH_4_^+^-N concentrations were higher during the wet season (48.16 ± 13.64 mg kg ⁻ ¹) compared to the dry season (32.32 ± 32.06 mg kg ⁻ ¹), but the difference was not significant (*p* > 0.05). Sediment pH showed minimal variation between the wet and dry seasons, with slightly higher values observed during the wet season (7.03 ± 0.19) compared to the dry season (6.85 ± 0.74). The DOC concentrations were significantly higher (*p* < 0.05) in the dry season (77.22 ± 5.22 mg kg ⁻ ¹) than in the wet season (23.36 ± 9.70 mg kg^-1^). Moreover, the values of SOM were slightly higher in the dry season (27.91 ± 6.54 g kg^-1^) compared to the wet season (22.92 ± 2.87 g kg^-1^). In addition, TN showed a slight increase in the dry season (1.81 ± 0.32 g kg^-1^) compared to the wet season (1.35 ± 0.12 g kg^-1^). The levels of NO_3_^-^-N remained similar between the wet (0.38 ± 0.27 mg kg^-1^) and dry seasons (0.40 ± 0.26 mg kg^-1^).

For GR and GRS, NH_4_^+^-N, NO_3_^-^-N and SOM amounts were higher during the wet season (NH_4_^+^-N: 54.09 ± 10.45 to 49.87 ± 11.60 mg kg^-1^; NO_3_^-^-N: 0.22 ± 0.15 to 0.35 ± 0.03 mg kg^-1^; SOM: 25.42 ± 2.79 to 25.58 ± 2.78 mg kg^-1^) compared to the dry season (NH_4_^+^-N: 24.11 ± 11.75 to 29.29 ± 6.78 mg kg^-1^; NO_3_^-^-N: 0.05 ± 0.03 to 0.11 ± 0.09 mg kg^-1^; SOM: 17.50 ± 1.84 to 21.19 ± 4.98 mg kg^-1^), with GR showing a significant difference (*p* < 0.05). The DOC amounts were significantly higher (*p* < 0.05) during the dry season (49.74 ± 4.38 to 67.02 ± 10.42 mg kg^-1^) compared to the wet season (12.80 ± 4.90 to 18.31 ± 9.45 mg kg^-1^). Sediment pH remained relatively stable between seasons, with slightly higher values observed in the dry season (6.49 ± 0.30 to 7.04 ± 0.67) than in the wet season (6.29 ± 0.23 to 6.79 ± 0.65). The TN values showed no consistent seasonal differences,

The PCA analysis of sediment phases in the rivers within the Jinjing Basin resulted in the creation of four new principal components that capture all of the dataset’s variability (Table 7). During the wet season, the first two PCs, each with eigenvalues greater than 1, accounted for 97.39% of the total variance (Table 7). The PC1 accounted for 77.75% of the variance and exhibited the closer positive correlations with NH_4_^+^-N, TN and SOM. A total variance of 19.64% was explained by PC2, which demonstrated stronger positive associations with DOC and pH. In the dry season, the first two PCs captured 92.73% of the total variance, but only PC1 had an eigenvalue more than 1 (Table 7). The PC1 explained 81.55% of the variance and demonstrated higher positive associations with nutrient content, SOM and DOC. In contrast, the PC2 accounted for 11.18% of the variance and showed stronger positive correlations with NO_3_^-^-N and pH.

**Table 7:** Weight and eigenanalysis of physicochemical parameters in the sediment across different rivers during (A) wet and (B) dry season in the principal component analysis.

A.				
Variable	PC1	PC2	PC3	PC4
NH_4_^ + ^-N	0.442	0.129	−0.710	0.347
NO_3_^-^-N	−0.429	0.330	−0.239	0.352
TN	0.446	0.113	0.601	0.646
SOM	0.459	0.085	−0.223	−0.119
DOC	0.234	0.789	0.165	−0.475
pH	−0.394	0.481	−0.018	0.314
Eigenvalue	4.665	1.179	0.128	0.029
Proportion	77.747	19.643	2.134	0.476
Cumulative	77.747	97.389	99.524	100.000
B.				
Variable	PC1	PC2	PC3	PC4
NH_4_^ + ^-N	0.444	−0.012	−0.348	0.046
NO_3_^-^-N	0.350	0.719	0.177	−0.561
TN	0.410	−0.244	0.684	0.057
SOM	0.440	−0.026	0.264	0.464
DOC	0.423	0.222	−0.498	0.379
pH	−0.374	0.611	0.250	0.567
Eigenvalue	4.893	0.671	0.296	0.141
Proportion	81.547	11.182	4.928	2.344
Cumulative	81.547	92.729	97.656	100.000

NH_4_^+^-N, ammonium nitrogen; NO_3_^-^-N, nitrate nitrogen; TN, total nitrogen; SOM, soil organic matter; DOC, dissolved organic carbon.

**Fig 5:**
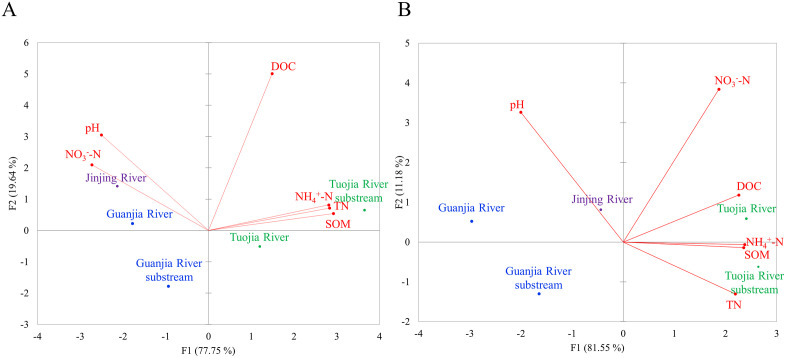
Principal Components Analysis (PCA) of physicochemical parameters in the sediment phases of rivers with Jinjing Basin during (A) the wet season and (B) the dry seasons. TN, total nitrogen, NO_3_^-^-N, nitrate nitrogen, NH_4_^+^-N, ammonium nitrogen, SOM, soil organic matter and DOC, dissolved organic carbon. Green colour indicates farmland, purple colour indicates residential area and blue colour indicates woodland.

**Fig 6:**
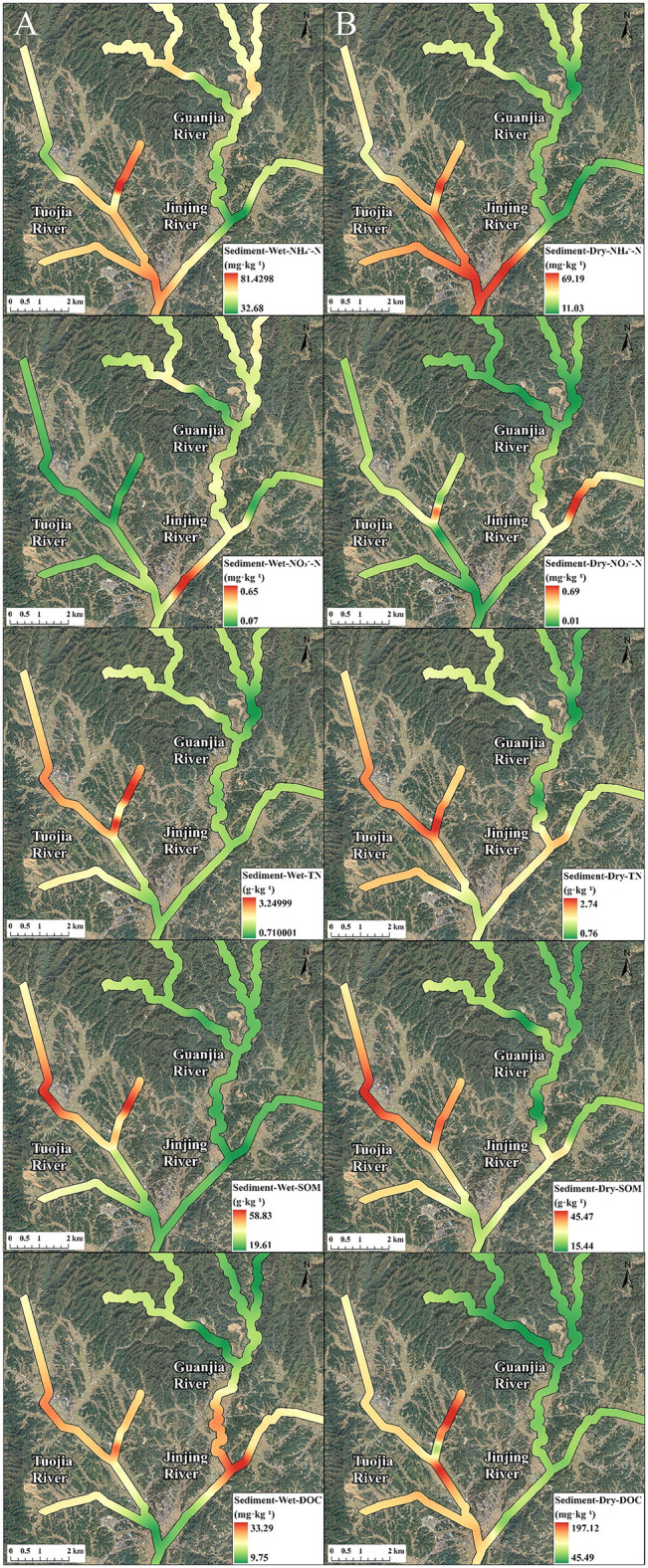
Inverse Distance Weighted (IDW) interpolation of physicochemical parameters in the sediment phase of rivers with Jinjing Basin during (A) the wet season and (B) the dry seasons. NH_4_^+^-N, ammonium nitrogen, NO_3_^-^-N, nitrate nitrogen, TN, total nitrogen, SOM, soil organic matter and DOC, dissolved organic carbon. Red colour indicates the highest value of the parameters, while dark green indicates the lowest value of the parameters.The base map was created by the authors using public domain data from the USGS National Map (http://viewer.nationalmap.gov/viewer/).

Similar to water phase, the PCA biplot showed that the sediment phases of the five rivers within the Jinjing Basin were clearly distinguished by the first two components, regardless of the season. (Fig 5) Furthermore, the rivers were grouped into two distinct clusters based on PC1. Overall, the first cluster, which included TR and TRS, was marked by higher nutrient content and elevated SOM and DOC values. In contrast, the second cluster, comprising GR, GRS, and JR, exhibited increased pH level. However, the second cluster displayed higher NO_3_ ⁻ -N level than the first cluster during the wet season. The IDW interpolation further corroborates the PCA findings by illustrating comparable spatial and seasonal trends, with TR and TRS characterized by greater nutrient, SOM and DOC levels (Fig 6).

## Discussion

### Impacts of spatial variations on water quality in the Jinjing Basin

The current analysis of water phase across different rivers in the Jinjing Basin indicated that the nutrient content in the TR and TRS is higher than in the other rivers (Table 2). A similar trend was observed in the sediment phase, where nutrient levels, SOM and DOC were also elevated in these rivers (Table 3). This is primarily attributed to the intensive agricultural activities in the region, leading to the classification of these rivers as farmland-dominated areas. This finding aligns with general observations worldwide, where agriculture regions tend to exhibit elevated nutrient levels in water bodies [12,14]. Farms heavily rely on fertilizers, pesticides and other chemicals, which eventually enter runoff and flow into the river system [15]. For instance, excessive use of nitrogen-based fertilizers and decomposition of organic matter release of NH_4_^+^-N into the soil, which eventually leaches into and pollutes the water system. Furthermore, vegetation and soil in the area absorb and retain some of these pollutants, further exacerbating the high nutrient concentrations in these rivers. Nevertheless, the present findings contrast with those of Ding et al. (2015) [6], who found that agricultural land contributed to less than 6% of the spatial variations in water quality in the Dongjiang Basin. This discrepancy could be attributed to the varying influence of agricultural land use on water quality, which largely depends on farming management practices and their intensity. For example, paddies are typically flooded after fertilizer application, allowing rice plants to absorb the nutrients. This practice reduces the impact on water quality in nearby streams [15,16].

Residential areas (JR) were identified as the second-largest source of pollutants in the Jinjing River Basin. This is evidenced by elevated nutrient concentrations in both water and sediment phases, along with high amount of SOM and DOC in the sediment. This pollution likely arises from point source, non-point sources or a combination of both. For example, the degradation of water quality may result from municipal wastewater discharges, chronic sewer leaks or illicit discharges. Additionally, some of the major cities such as Dongguan and Huizhou have failed to adequately treat sewage, contributing to the deterioration of water quality in the rivers [6]. Furthermore, the expansion of residential areas increases impervious surfaces, which enhance storm water flows and runoff volumes, indirectly elevating nutrient concentrations in the rivers.

Guanjia river and GRS are classified as woodlands, characterized by relatively stable topography and hydrology. Thus, the water flow in these areas remains steady with minimal fluctuations [17]. This is due to the dense vegetation cover in these areas, which aids in stabilizing the soil, absorbing excess amount of pollutants and minimizing nutrient loss from the soil. Furthermore, woodland serves as a microbiological nutrient detention zone, where processes such as microbial assimilation and denitrification frequently occur [4]. Therefore, the woodland effectively decrease the nutrient loads transported into the river via surface runoff. This is evidenced by the lowest nutrient concentrations in both the water and sediment phase of GR and GRS. These findings are further supported by reduced amounts of SOM and DOC in the sediment phase (Table 2 and 3). The critical role of woodland in preserving high water quality parameters must not be underestimated.

### Impacts of seasonal variations on water quality in the Jinjing Basin

The water quality of a water body is strongly influenced by the climate of a region [3,5]. Additionally, seasonal variations have been shown to affect the relationship between land use and water quality [18,19]. Although the Jinjing Basin experiences four distinct seasons [7], the subtropical climate of this area is characterized by a clear distinction between the dry and wet seasons. These seasonal variations can substantial influence hydrological processes such as runoff, precipitation, interception, and abstraction, which in turn affect river flow. Consequently, they contribute to varying levels of sediment and nutrient across different seasons [2,19]. Overall, the water phase of the rivers in the Jinjing Basin exhibited higher nutrient concentrations and Eh values during the wet season compared to the dry season, except for NH_4_^+^-N, pH, and DO levels, which were lower (Table 4). In contrast, the sediment phase illustrated an opposite trend: NH_4_^+^-N amount was higher, NO_3_^-^-N amount was lower and TN amount remained consistent during the wet season compared to the dry season (Table 6). The discrepancy between water and sediment phase within the same season could be explained by the distinct biogeochemical processes governing nutrient dynamics, such as the varying rates of organic matter decomposition, nitrogen transformation pathways (e.g., nitrification and denitrification) and sediment-water exchange influenced by hydrological conditions and microbial activity [20,21].

In the water phase, higher pH and DO levels observed in every rivers during the dry season, particularly in GR, GRS, and TRS, reflect the impact of reduced water flow and increased photosynthetic activity. Lower flow rates during the dry season often result in prolonged water retention times, allowing for greater biological activity, including photosynthesis by aquatic plants and algae, which can increase pH and DO. In contrast, the wet season’s lower pH and DO levels may result from dilution effects due to increased rainfall and runoff, which introduce organic matter and reduce oxygen availability through enhanced microbial respiration [22,23]. The strong correlation between pH and DO levels in the dry season, as indicated by PCA results, further supports the influence of seasonal variation on the biological processes within the rivers. During the wet season, NO_3_^-^-N and TN concentrations were higher in most rivers, particularly in JR, TR and TRS. This pattern indicates increased nutrient inputs from surface runoff, especially from agricultural and residential areas. The elevated nutrient levels during the wet season highlight the vulnerability of these rivers to non-point source pollution, which is exacerbated by rainfall-driven transport of fertilizers, animal waste, and other nutrient-rich materials [24]. A similar trend was observed in GR, where the wet season likely intensified surface runoff, washing organic matter and decomposing vegetation from the surrounding woodland into the river. The breakdown of this organic material releases nitrates, contributing to the elevated NO_3_^-^-N concentrations. Additionally, higher flow rates during the wet season may enhance the leaching of nitrogen compounds from the soil in the woodland areas, further increasing NO_3_^-^-N levels in the water [24,25]. For NH_4_^+^-N, a substantial increase in concentrations during the dry season compared to the wet season was identified in GR and GRS. One possible explanation for this is that reduced water availability in woodlands during the dry season leads to lower oxygen concentrations, which in turn slows down nitrification rates, resulting in the accumulation of higher NH_4_^+^-N concentrations [26]. This explanation is further corroborated by the lower Eh values during the dry season, indicating more reducing conditions that can favor processes like denitrification and the release of NH_4_^+^-N and other reduced compounds [27].

For sediment phase, higher NH_4_^+^-N and SOM amounts were observed during the wet season in most rivers. This could be attributed to increased leaching and runoff during periods of heavy rainfall, which introduces ammonium-rich fertilizers and organic materials into the sediment [24]. A similar mechanism explains the relatively higher NO_3_^-^-N levels observed in GR and GRS during the wet season, where increased hydrological flow facilitates the mobilization of nitrate from terrestrial sources into the sediment. Conversely, higher NO_3_^-^-N concentrations in the sediment during the dry season, especially in TR and TRS, suggest nitrification processes in aerobic conditions, which can also elevate NO_3_^-^-N in the water [27]. The lack of a specific trend in TN values observed in this study could be attributed to the variability in nitrogen inputs (e.g., surface runoff or atmospheric deposition) and outputs (nitrification, denitrification or plant uptake), which fluctuate depending on seasonal conditions. However, no significant differences (*p* > 0.05) in TN between the wet and dry seasons reflects the role of sediment as a stable sink for nitrogen over time, effectively buffering the impacts of seasonal fluctuations [28]. Meanwhile, sediment pH showed minimal variability across seasons, suggesting the buffering capacity of the sediments and the stable geochemical environment. Slightly higher pH values observed during the dry season may result from reduced acid inputs from precipitation and increased carbonate dissolution under lower water flow conditions [29]. The DOC concentrations exhibited significant differences (*p* < 0.05) across season, with higher levels consistently observed during the dry season. This trend can be attributed to reduced dilution by surface runoff and enhanced decomposition of organic matter under aerobic conditions. The elevated DOC in the dry season may also indicate higher microbial processing of SOM, releasing soluble organic compounds into the sediment [20,27].

A comprehensive understanding of river water quality involves more than examining individual physicochemical parameters but it also requires analyzing their interrelationships. This is because these parameters are interconnected via biogeochemical processes. For example, nutrient enrichment can stimulate microbial respiration, reducing oxygen and Eh value, which in turn affects nitrogen transformations between NH_4_^+^-N and NO_3_ ⁻ -N. Meanwhile, increased levels of DOC and SOM can lead to higher oxygen demand and affect redox-sensitive nutrient cycling. Likewise, pH and DO are also regulated by photosynthetic activity and biological oxygen demand, which fluctuate with nutrient and organic matter concentrations. These interactions become especially dynamic across seasons, as hydrological and biological shifts can modulate the balance between photosynthesis and respiration, oxidation and reduction as well as the uptake and release of nutrients (Figs 3 and 5). Hence, studying the interplay among these parameters provides a more holistic understanding of aquatic ecosystem functioning and the cumulative impacts of land use and climate variability.

In addition to hydrological and biological influences, geochemical processes are important in regulating the seasonal behavior of nutrient parameters. During the wet season, increased surface runoff delivers organic matter and nutrients into the river system, leading to higher levels of SOM and nutrients particularly NH_4_^+^-N in the sediment phase (Table 6). Furthermore, ammonification activity appears to contribute to the elevated NH_4_^+^-N levels, where microbial decomposition of organic matter releases NH_4_^+^-N. Conversely, the lower NH_4_^+^-N levels observed during the dry season may result from increased microbial activity, where localized aerobic microsites in the sediment surface promote nitrification, converting NH_4_^+^ to NO_3_^-^ Moreover, the decline in sediment NH_4_^⁺^-N may also influenced by decreased organic matter input and possible desorption or diffusion of NH_4_^+^-N into the water column. Under reducing conditions (lower Eh value during dry season), organic matter decomposition may elevate, resulting in the increased DOC concentrations observed in the sediment during the dry season. This can be ascribed to microbial mineralization of SOM and desorption of organic compounds from sediment particles, which improve DOC release. These processes may also affect the transformation and mobility of nitrogen compounds within the sediment environment [30]. Sediment pH showed minimal seasonal variation, possibly buffered by carbonate dissolution or mineral weathering, especially in forested regions. These results highlight the role of redox processes, organic matter decomposition and sorption-desorption process in regulating nutrient dynamics in river sediments.

### Identification of pollution sources in Jinjing Basin

The PCA provided critical insights into the sources and distribution of pollution within the Jinjing Basin across wet and dry seasons. The separation of rivers into two clusters based on PC1 (water and sediment phases) highlights the influence of land use on pollution profiles (Figs 3 and 5). In the water phase, nutrient-related variables such as NH_4_^⁺^-N, NO_3_ ⁻ -N, TN, and TP dominated PC1 for both wet and dry seasons underscores the major contribution of nutrient pollution in the rivers. A similar pattern was identified in the sediment phase, where the nutrient parameters (NH_4_^⁺^-N, TN) and organic matter indicators (SOM, DOC) were prevalent in PC1 for both wet and dry seasons, suggesting nutrient enrichment strongly impacts sediment quality. The elevated nutrient levels observed in the first cluster (TR and TRS), characterized by agricultural land use, strongly suggest agricultural runoff and other anthropogenic inputs are the primary sources of pollution. Moreover, this cluster based on water phase displayed higher Eh values, proposing oxidation-reduction conditions that facilitate the release and mobility of nitrogen and phosphorus species. Additionally, elevated SOM and DOC values further confirm the substantial organic and nutrient inputs from agricultural activities, which become stabilized in the sediment matrix [31]. Therefore, PCA biplot identified these areas surrounding TR and TRS as pollution hotspot. Meanwhile, the second cluster based on water phase, comprising GR and GRS (woodland) and JR (residential area) demonstrated higher pH and DO levels. These characteristics likely reflect reduced nutrient inputs and the natural buffering capacity of the water in these areas. Yet, higher nutrient levels in JR, suggest localized source of pollution, such as organic matter decomposition in residential zones. Similarly, higher pH values were found in the sediment phase of the second cluster, which possibly reflect natural buffering from carbonate materials in woodland and residential zones [13,29].

The seasonal analysis revealed distinct differences in physicochemical dynamics of the Jinjing Basin. During the wet season, PC1 based on water phase explained a larger proportion of the total variance (77.11%), driven by high nutrient inputs possibly from agricultural runoff due to precipitation (Table 5). This finding aligns with the heightened transport of pollutants during rainfall, which accelerates the movement of dissolved and particulate nutrients into water bodies [24]. In contrast, the dry season exhibited lower nutrient contributions to PC1 (65.44%) and higher contributions to PC2 (24.56%), with pH and DO emerging as key variables. This seasonal shift suggests that lower runoff during the dry season reduces organic loading, enabling higher DO levels through enhanced photosynthetic activity in clearer waters. Additionally, the higher pH observed in the dry season may be attributed to a reduced influx of acidic substances from runoff, combined with biological activities such as algal photosynthesis [32,33]. In the sediment phase, during the wet season, PC2 captures a significant portion of the variance (19.64%) and is positively associated with DOC and pH (Table 7). This relationship suggests that wet season hydrology facilitates the transport of dissolved organic carbon and influences the buffering capacity of sediments [34]. In contrast, during the dry season, PC2 accounted for a smaller portion of the variance (11.18%) and was characterized by higher correlations with NO3 ⁻ -N and pH. This shift reflects reduced nutrient loading from surface runoff but emphasizes nitrate accumulation in sediment, possibly through nitrification processes under aerobic conditions. However, decreased runoff in the dry season allows natural processes such as stabilization of organic matter in sediments and photosynthesis to dominate [35,36].

In addition to direct pollution inputs, the mobility and fate of nutrients in both the water and sediment are also influenced by underlying physicochemical conditions, particularly pH, EC and the presence of coexisting ions. For instance, pH governs the chemical form and bioavailability of nutrients of nitrogen compounds such as NH_4_^+^-N, which can shift toward NO_3_^-^-N at higher pH, while nitrification processes are reduced under acidic conditions [37]. In this study, the relatively stable and near neutral pH indicate moderate buffering, supporting the coexistence of both NH_4_^+^-N and NO_3_^-^_-_N depending on microbial activity and redox state. Electrical conductivity represents the total ionic strength of the river system and is often higher in farmland streams (e.g., TR and TRS) as a result of fertilizer runoff. Higher EC can facilitate the release of nutrients from sediments and increase their mobility, especially in the presence of competing cations such as Na^+^, K^+^, Ca^2+^ and Mg^2+^ introduced through agricultural or domestic sources [9]. These factors likely drive the nutrient enrichment observed in rivers near to farmland as shown in the multivariate analysis (Figs 3 and 5).

The water and sediment quality patterns observed in the Jinjing Basin are regulated by both human activities and natural processes. Agricultural areas (TR and TRS) mainly reflect anthropogenic influences, including the excessive fertilizer application, soil disturbance and livestock waste, which lead to high levels of NH_4_^+^-N, TN, and SOM. Similarly, residential areas (JR) show anthropogenic impacts through domestic wastewater discharge and increased runoff from impervious surfaces that enhance the nutrient enrichment in the aquatic system. On the other hand, woodland areas (GR and GRS) are predominantly shaped by natural processes. The dense vegetation canopy decreases erosion and surface runoff, while microbial assimilation, plant uptake and denitrification naturally reduce nutrient levels in both water and sediment. These areas also show more stable redox conditions and buffering capacity, maintaining higher DO and pH levels. This highlights the natural retention processes within forested regions contrast to the human-driven nutrient enrichment in agricultural and urban areas.

Although this study did not directly measure microbial indicators such as total coliform *Escherichia coli*, relevant findings from the same region offers strong evidence of microbial contamination in residential areas. For instance, Asfi et al. (2023) [38] reported exceptionally high levels of total coliform (up to 162 x 10^3^ CFU mL^-1^) and *E. coli* (up to 64 x 10^3^ CFU mL^-1^) in gross pollutant traps and wetland inlets situated near high-density residential areas in Putrajaya, Malaysia. In addition, *E. coli* concentrations were found to be more abundant during the dry season, which aligns with the present findings of higher SOM and DOC levels in the same period. These findings indicate that the domestic sewage from residential areas could be a contributing factor to the observed microbial contamination pattern, especially under conditions that promote organic matter accumulation and the survival of microbes.

Elevated levels of nitrogenous compounds (especially NH_4_^⁺^-N and NO_3_⁻-N) and organic matter (DOC and SOM) identified in farmland-impacted rivers like TR and TRS have important environmental implications for aquatic ecosystems. For example, higher concentrations of NH_4_^⁺^-N can be harmful to aquatic life, particularly under high pH and temperature, where NH_4_^⁺^-N shift to unionized ammonia (NH_3_), which is more toxic. Similarly, excessive NO_3_⁻-N can contribute to eutrophication, stimulating algal blooms and subsequently lead to oxygen depletion during algal decomposition [37]. On the other hand, higher concentrations of DOC and SOM can enhance microbial respiration, resulting in hypoxia or anoxia, which further disrupt aquatic ecosystems. These changes can decrease biodiversity, impair ecosystem functions and alter nutrient cycling processes. If left unmanaged, continued nutrient loading from agriculture and residential areas could lead to long-term degradation of river health, harm aquatic organisms, compromise water quality as well as decrease the availability of clean water for human consumption [39].

Beyond immediate ecological effects, these contaminants may also pose long-term risks through bioaccumulation and potential biomagnification within aquatic food webs. For example, excessive nitrogen compounds (e.g., NH_4_^⁺^-N and NO_3_⁻-N) can be assimilated by primary producers, entering the food web and potentially affecting the higher trophic levels through consumption. Prolonged exposure may impair growth, reproduction and metabolism functions in aquatic life, particularly in environments already suffered by eutrophication or oxygen depletion [40]. In addition to nutrients, agricultural runoff or domestic wastewater may introduce trace metals and persistent organic pollutants, which can accumulate in aquatic organisms and biomagnify through the food web, subsequently posing ecological and public health risks [41].

### Management implications for Jinjing Basin

Rivers in the Jinjing Basin, particularly TR and TRS, demonstrated clear signs of nitrogen contamination and borderline phosphate levels, based on the drinking water quality standard of China [42]. Current findings highlight rivers near farmland, especially TR and TRS contribute the most substantially to the deterioration of water quality in the basin. Hence, prioritizing the restoration and management of these two rivers is essential for improving overall water quality. Implementing targeted and effective management measurements are direly needed to address this issue. Firstly, adopting a range of best management practices (BMPs) on agricultural farms can considerably improve farming practices. The BMPs include applying fertilizer at the appropriate rate and timing, utilizing advanced soil nitrogen testing methods, adopting crop rotation systems, incorporating legume crops and implementing crop residue management along with the use of cover crops [43,44]. For instance, these BMPs can help reduce excessive “insurance” applications of nitrogen fertilizer, potentially lowering nitrogen runoff into water bodies by an estimated 0.9 to 1.4 million metric tons annually [43]. Secondly, riparian zones and wetlands should be actively promoted due to their crucial role in intercepting groundwater and surface water flowing laterally from farmlands. These areas serve as natural buffers between agricultural uplands and nearby water bodies. Research suggests that riparian forests and restored or created wetlands, when designed ecological engineering principles, can decrease nitrogen levels by approximately 4 g N m^-2^ per year and 10−20 g N m^-2^ per year, respectively [43]. Thirdly, aquatic plants play a vital role in improving water quality by removing excess nutrients, such as nitrogen and phosphorus, from water bodies. For example, duckweed has been shown to effectively remove nutrients from wastewater. Additionally, these plants can serve as a valuable resource, either as a food source for animals or as a natural fertilizer in agriculture, further enhancing their utility and sustainability [45]. Similarly, algae are well-known for their effectiveness in eliminating excessive nutrients from water. For instance, floating algae are frequently utilized in ponds to absorb nutrients from contaminated streams [46]. Like aquatic plants, algae can also be repurposed as animal feed or biofertilizers in agricultural sector after accumulating these nutrients [45]. The timing of implementing aquatic plants and algae plays a pivotal role in reducing nutrient pollution. Based on the current findings, it is strongly recommended to cultivate these plants during the wet season to effectively mitigate nutrient pollution, particularly in TR and TRS within Jinjing Basin. Meanwhile, the present study reveals that the river (JR) near residential area is also polluted with nutrients. Remarkable and persistent sources of such pollutants include illicit discharge connections, leaking sewer systems and failing septic systems. To address this issue effectively, the implementation of advanced water treatment plants, as well as robust sewage and water purification infrastructure, is essential in mitigating pollution from residential areas [6].

## Conclusions

This study provides valuable insights into the seasonal and spatial variability of physicochemical parameters in water and sediment across rivers within Jinjing Basin. Rivers near farmland, such as TR and TRS, consistently exhibited higher nutrient concentrations such as NH_4_^+^-N and TN in the water phase, suggesting potential anthropogenic influences or natural enrichment processes within these rivers. Conversely, GR and GRS located to adjacent to woodland areas, displayed lower nutrient levels alongside higher DO and pH values, reflecting healthier water quality conditions and a greater capacity to support aquatic ecosystems. Seasonal differences influenced nutrient dynamics, with the wet season showing elevated nutrient levels in both water and sediment phases due to the influence of surface runoff and increased water flow on nutrient loading. The integration of water and sediment phase highlighted the interplay between surface and subsurface processes across season: nutrient enrichment occurred during the wet season through runoff and subsequent accumulation in sediments, whereas the dry season exhibited higher sign of organic matter decomposition (higher DOC value) under reducing conditions. Principal component analysis further emphasized the seasonal and spatial segregation of physicochemical properties, particularly the clustering of rivers with high nutrient contents and the divergence of others with differing DOC or pH characteristics. Nutrient pollution from farmland streams emerged as the dominant factor influencing biogeochemical patterns in both water and sediment. These findings highlight the importance of monitoring and managing both water and sediment phases to maintain aquatic ecosystem health. In line with Sustainable Development Goal 6 (Clean Water and Sanitation), this study reinforces the urgent call for river rejuvenation initiatives. In the context of China, frameworks such as the Water Pollution Prevention and Control Law and Hunan’s Xiangjiang River Basin regulations provide a strong legislative foundation to address non-point source pollution and promote river health restoration. To support these policy efforts, national initiatives should integrate sediment-phase assessments alongside conventional water monitoring. Evidence-based monitoring and targeted management strategies are needed to decrease nutrient inputs, particularly in high-risk areas such as TR and TRS. Future research should focus on elucidating the underlying biogeochemical processes and exploring effective mitigation strategies such as the construction of wetlands, riparian buffer enhancements and other nature-based or engineered solutions to reduce nutrient pollution and enhance the resilience of subtropical riverine ecosystems to seasonal variations.

## Supporting information

S1 Table Raw data of the study. (XLSX)
